# A comprehensive framework for operationalizing structural racism in health research: The association between mass incarceration of Black people in the U.S. and adverse birth outcomes

**DOI:** 10.1016/j.ssmph.2022.101225

**Published:** 2022-09-08

**Authors:** Anders Larrabee Sonderlund, Mia Charifson, Robin Ortiz, Maria Khan, Antoinette Schoenthaler, Natasha J. Williams

**Affiliations:** a Center for Healthful Behavior Change, Institute for Excellence in Health Equity, NYU Grossman School of Medicine, USA; b Research Unit of General Practice, Department of Public Health, University of Southern Denmark, Denmark; c Department of Population Health, NYU Grossman School of Medicine, USA; d Vilcek Institute of Graduate Biomedical Sciences, NYU Grossman School of Medicine, USA; e Department of Pediatrics, NYU Grossman School of Medicine, USA

**Keywords:** Adverse birth outcomes, Incarceration, Community health outcomes, Structural racism, Racial/ethnic health disparities

## Abstract

Structural racism represents a key determinant of the racial health disparities that has characterized the U.S. population throughout its existence. While this reality has recently begun to gain increasing acknowledgment and acceptance within the health sciences, there are still considerable challenges related to defining the concept of structural racism and operationalizing it in empirical study. In this paper, building on the existing evidence base, we propose a comprehensive framework that centers structural racism in terms of its historical roots and continued manifestation in most domains of society, and offer solutions for the study of this phenomenon and the pathways that connect it to population-level health disparities. We showcase our framework by applying it to the known link between spatial and racialized clustering of incarceration – a previously cited representation of structural racism – and disparities in adverse birth outcomes. Through this process we hypothesize pathways that focus on social cohesion and community-level chronic stress, community crime and police victimization, as well as infrastructural community disinvestment. First, we contextualize these mechanisms within the relevant extant literature. Then, we make recommendations for future empirical pathway analyses. Finally, we identify key areas for policy, community, and individual-level interventions that target the impact of concentrated incarceration on birth outcomes among Black people in the U.S.

## 1 Introduction

Black people in the United States (U.S.) are heavily overrepresented on a range of negative health outcomes. For example, while heart disease is slightly more prevalent in non-Hispanic White (hereafter referred to as White) compared to Black populations (11.5% versus. 9.5%), the associated death rate is nearly 20% higher in the latter (CDC and Health, 2019). Similarly, Black people have a 12% increased risk of death from cancer compared to Whites. For breast and prostate cancer, the relative risk of death increases to 30% and 100%, respectively (CDC, 2019). Comparable disparities exist for a range of health-risk factors and other chronic diseases, including stress, diabetes, high blood pressure, and obesity (Merai et al., 2016; Wall et al., 2018).

A robust and growing body of research implicates structural racism as a key determinant of many of these health disparities (Bailey et al., 2017, 2021). Different from interpersonal racism, which occurs at the individual level, structural racism is defined in terms of its societal nature and systemic, multi-level manifestation (Bailey et al., 2017, 2021; Feagin, 2020; Massey & Brodmann, 2014). For example, the practice of redlining – an oft-cited instance of structural racism – relates primarily to discriminatory zoning laws and mortgage lending that disproportionately affect Black people in the U.S. These practices profoundly restrict housing opportunities and socio-economic mobility among residents, effectively perpetuating de facto racial and economic residential segregation (Massey & Brodmann, 2014; Massey & Fischer, 2000). This has myriad downstream social and health-related consequences for the communities in these areas, including constrained access to quality health care and housing, education and employment (Rothstein, 2017). Thus, as highlighted in this example, structural racism denotes the racist biases that permeate the mechanics of social institutions (e.g., housing and finance in the case of redlining) and enable systemic racial discrimination within and across these institutions. In turn, this results in systematic disadvantage, marginalization, and oppression of Black people – often in terms of restriction of rights and access to public and private resources (e.g., health care, insurance) and typically with severe repercussions for both physical and mental health (Adkins-Jackson et al., 2021; Bailey et al., 2017, 2021; Jahn, 2021).

In order to reflect its ubiquity, the definition of structural racism is purposely broad and multifaceted. This complexity, however, becomes problematic when moving from a conceptual understanding of the phenomenon to an empirical examination of the specific pathways that lead to health outcomes. To date, most research on structural racism centers on a narrow set of proxies – mainly inequities in housing, economy, criminal justice – and the individual association with population health. Only few studies have focused on other domains (e.g., health care, education) and even fewer have tested potential mediators of the impact on health (Bailey et al., 2017, 2021; Gravlee, 2020; Janevic et al., 2020; Krieger et al., 2020; Williams & Collins, 2001). However, elucidating the multiple interacting systems and pathways through which structural racism affects health outcomes is crucial for effective intervention development and dissemination. While recent efforts in the social and health sciences to steer empirical study toward this end are steadily gaining momentum (Alson et al., 2021; Bailey et al., 2021a, 2021b; Churchwell et al., 2020; Gee & Ford, 2011), operationalizing structural racism and mapping the pathways to health outcomes remains a significant challenge (Groos et al., 2018; Jahn, 2021). In this paper, we propose a conceptual framework of the primary societal systems in which structural racism manifests to produce racial health disparities among Black people. Our framework is designed to serve as a general blueprint to advance future theory and help focus empirical investigation into the numerous individual and interacting processes that underpin the association between structural racism and population-health outcomes. In the following sections, we present the core features of our framework (Section 1.1), after which we illustrate its utility by applying it to the known association between racialized and spatially clustered mass incarceration (our chosen example and operationalization of structural racism) and disparities in adverse birth outcomes (Section 2). Specifically, given the general lack of knowledge about the mechanics of this relationship, we use our model to identify the pathways that lead from racialized mass incarceration to community-level disparities in adverse birth outcomes.

### 1.1 Conceptual framework: a starting point

**Fig. 1:**
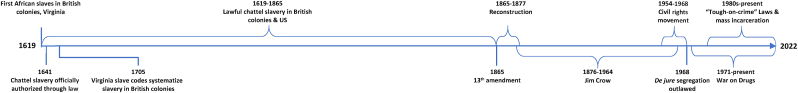
Timeline of key events in U.S. history related to the systematic oppression of Black Americans.

Contextualizing our framework historically, we emphasize the importance of acknowledging and understanding the root cause of structural racism in the U.S. This relates specifically to the White supremacist ideologies, values, and practices (e.g., enslavement of African people) that emerged in Europe in the 1400s and transmitted to the Thirteen British Colonies and the nascent U.S. society. The timeline (Fig. 1) shows how these racist ideologies have survived throughout American history, initially manifesting overtly as chattel slavery and Jim Crow and continuing to reemerge in exceedingly insidious, but devastating incarnations (e.g., de facto residential segregation, mass incarceration). This history of racist ideas and their perpetuation in the U.S. has been discussed extensively by numerous scholars – notably DuBois (DuBois, 1903), Kendi (Kendi, 2016), and Painter (Painter, 2010) – as the root cause of the persistent White-centric bias that characterizes the unequal distribution of resources and opportunities for social and economic advancement. We emphasize that an acknowledgment of this background is necessary for a deeper understanding of the health and well-being of Black people in the U. S. We also contend that an omission of this history contributes to the perpetuation and reinforcement of structural racism and pathology of Black people in the U.S. Thus, the historical context is provided not only to anchor our theoretical approach, but also to ground and enhance future health equity research.

**Fig. 2:**
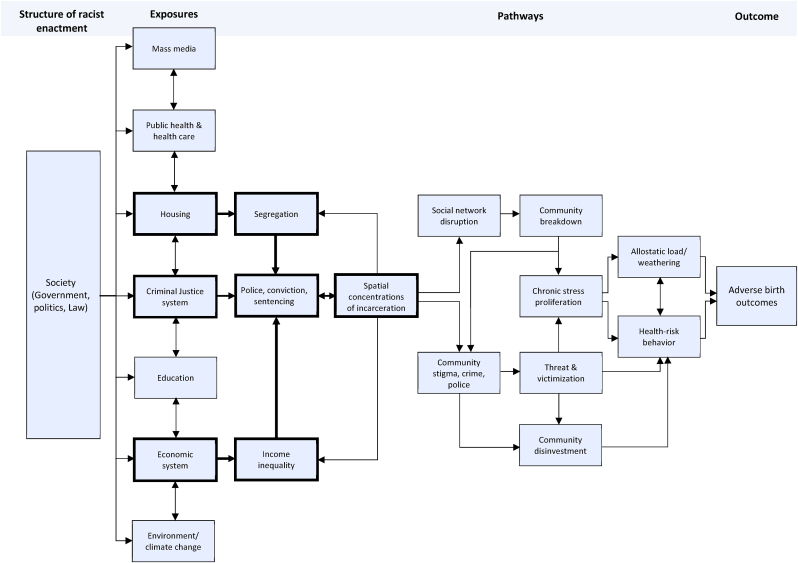
The hypothesized pathways by which structural racism (exposure variable) contributes to adverse birth outcomes among Black pregnant persons.

In developing our framework, we build on a broad range of previous work in the area of racism, discrimination, and health. While we elaborate on past conceptualizations of the broader construct of structural racism (Bailey et al., 2017; Williams & Mohammed, 2013; Yearby, 2020), we also draw on specific theories on individual pathways by which racism may impact on health outcomes. These include, for instance, the biopsychosocial model of racism (Clark et al., 1999; Geronimus, 1992), environmental racism (Bryant & Mohai, 2019), and intersectionality (Viruell-Fuentes, Miranda, & Abdulrahim, 2012). We argue that the theoretical comprehensiveness combined with the operational detail of our framework facilitates its adaptability and implementation in both applied and theoretical research. In this way, our framework serves to integrate and extend existing evidence, which often relies on a generalized conceptualization of structural racism limited to a specific outcome (Martinez et al., 2021) (Russo et al., 2021). As depicted in Fig. 2, our framework comprises two core sections within which pathways to particular outcomes of interest can be contextualized and hypothesized: *Structure of Racist Enactment* and *Exposures*.

*Structure of racist enactment* refers to the concrete enactment of the racist philosophies inherent in U.S. society. This relates primarily to the administration and distribution of resources and opportunity through discriminatory legislation, policy, and law. The structure of racist enactment may best be defined as the ideological foundation upon which society is built and from which the hierarchical and unequal social, political, and legislative reality that we inhabit and experience continues to be produced (Bonilla-Silva, 1997). In line with current thinking, we posit that the racist elements inherent in this structure manifest in multiple societal systems through which racial disparities in health are engendered along many distinct, but often intersecting pathways (Bailey et al., 2021a, 2021b; Gee & Hicken, 2021; Hicken et al., 2021; Massey & Brodmann, 2014; Rothstein, 2017). Specifically, the *Exposures* column of our framework depicts seven systems which we, based on our critical review of the evidence, deem as central to the association between structural racism and health inequities. As noted above, in the context of health and well-being, the literature has focused most prominently on residential segregation (housing system) (Landrine et al., 2017; Rothstein, 2017; Williams & Collins, 2001; Zhou, Bemanian, & Beyer, 2017), racial and ethnic inequalities in wealth and income (economic system) (Dowd, Simanek, & Aiello, 2009; Dwyer, 2010; Fleisch Marcus et al., 2017; Massey, 2012), racialized policing and incarceration (criminal justice system) (Alexander, 2010, p. 377; Dobbie, Goldin, & Yang, 2018; Dumont et al., 2013; Wildeman & Wang, 2017), as well as environmental domains (e.g., disparities in life-course exposure to toxic waste dumping sites, chemical contaminants, air pollution) (Bailey et al., 2021a, 2021b; Gutschow et al., 2021; Hammer, 2019; Kaufman & Hajat, 2021; Lopez-Littleton & Sampson, 2020; Nigra, 2020; Perry et al., 2021; Pulido, 2000). Additionally, a relatively small but increasing number of studies reports similar indications of structural racism and associated health consequences in other domains as well, including inequalities in access to quality education (Crutchfield, Phillippo, & Frey, 2020; Lloyd, Carlson, & Logan, 2021; Lucey & Saguil, 2020; Merolla & Jackson, 2019) and health care (Williams & Mohammed, 2013; Yearby, 2018, 2020), and discriminatory mass media representation (e.g., perpetuating negative racial stereotypes as well as social invisibility of minority populations and issues of disproportionate relevance to these populations) (Birk, Gill, Heer, & De-Islamizing Sikhaphobia, 2015; Leavitt et al., 2015; Nairn et al., 2006; Rosino & Hughey, 2018; Sesko & Biernat, 2010). While these exposures are often conceptualized and studied as distinct and separate, in our framework (Fig. 2) we contend that they are often complex and interdependent. Generating testable hypotheses based on our framework would thus begin at the *Exposures* column.

## 2 A case study: mass incarceration of Black people as a measure of structural racism

To illustrate our model, we have chosen to focus on mass incarceration of Black people as a manifestation of structural racism. On any given day, U.S. state and federal prisons contain approximately 2.3 million prisoners – the highest per capita rate of incarceration in the world. Additionally, there are currently an added 4.9 million Americans with a history of incarceration (Sawyer, 2020a). Within these populations, there are major disparities on key dimensions of race/ethnicity, income, and place of residence. Black people make up approximately 13% of the U.S. population, but account for over 40% of the incarcerated population. By contrast, White people represent 57% of the general population, but only 39% of the prison population (Sawyer, 2020b). This represents an inequity where Black men are nearly six times as likely as White men to be incarcerated (Visualizing the racial disparities in, 2020). Further, according to Lee and colleagues, nearly half (44%) of all Black women in the U.S. have a family member who is incarcerated. By comparison, only 12% of White women have family in prison (Lee et al., 2015). In terms of economic inequalities in the incarcerated population, the median annual pre-incarceration income for Black and White male prisoners in 2014 was $17,625 and $21,975, respectively (Rabuy & Daniel, 2015).

**Fig. 3:**
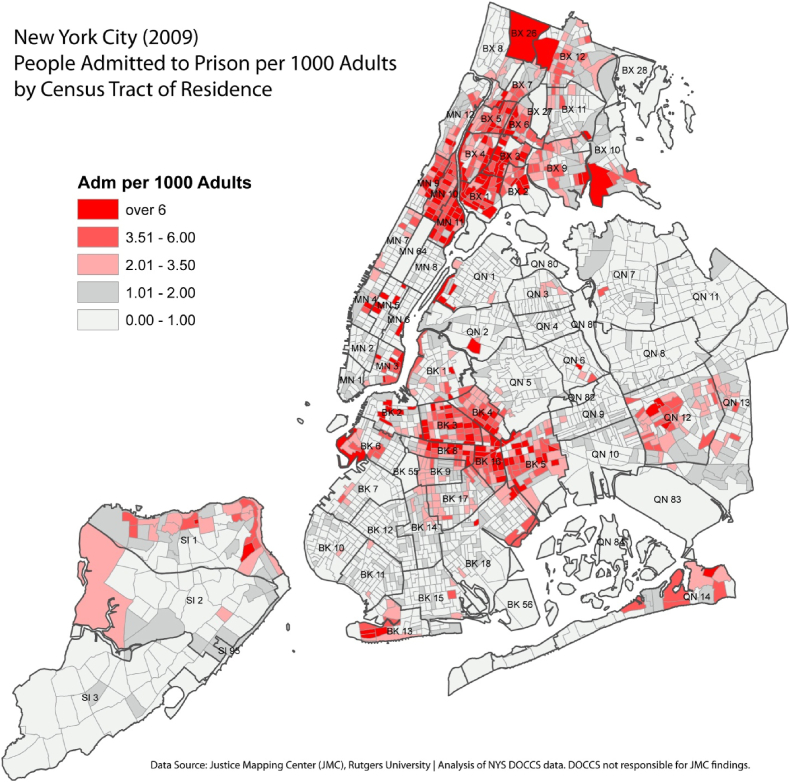
Spatial clusters of incarceration in New York City (2009).

Driving these disparities in incarceration rates, the evidence indicates that the systems of housing, economy, and criminal justice converge to position Black populations at a particularly increased risk of incarceration. Specifically, because housing in the U.S. is often racially and economically segregated, the concentrated incarceration rates in low-income Black populations also cluster geographically and disproportionately in low-income majority-Black neighborhoods. For example, in New York City, spatial incarceration rates correlate strongly with race and SES, with extreme concentrations of incarceration clustering in low-income majority-Black census tracts (notably in the Bronx and the north-eastern parts of Brooklyn, see Fig. 3). Similar patterns are evident in cities across the U.S. (Vera. Incarceration Trends, 2020). This phenomenon has resulted from decades of both *de jure* (during Jim Crow) and de facto (e.g., redlining) residential segregation, where Black people, particularly low-income, are funneled into underserved spaces in which social and economic mobility is all but impossible (Massey & Brodmann, 2014). In addition to pervasive social disadvantage, these areas are often also overpoliced, driving up arrest rates among residents who, given various tough-on-crime laws (e.g., stop-question-and-frisk zones, ‘reasonable suspicion’), are typically apprehended for misdemeanors or other minor non-violent felonies (Jahn, 2020; 2020aSawyer). Following disparities in arrest, the systems of bail, legal representation, plea bargains, and judge sentencing show additional racial biases in terms of conviction rate and harshness in sentencing (Alexander, 2010, p. 377; Commission, 2018).

### 2.1 The population-health effects of incarceration: adverse birth outcomes

The negative health effects of serving time in jail or prison are pervasive. Compared to the general population, people with a history of incarceration have a higher likelihood of infectious disease, stress-related illnesses, cardiovascular diseases, and cancer (Binswanger et al., 2007; Massoglia, 2008; Rosen, Schoenbach, & Wohl, 2008). Patterson (Patterson, 2013) found an added two-year decrease in life expectancy for each year spent in prison. Importantly, these health effects do not stop at the individual, but also spill over to affect the individual's social networks and the wider community in which they exist. For example, paternal incarceration has been associated with increased risk of obesity (Roettger & Boardman, 2012) and mortality (Wildeman, 2012) in dependent children. Mothers of imprisoned adult children and women with incarcerated spouses are significantly more likely than the general population to have obesity, have had a heart attack or stroke, contract sexually transmitted infections, and be in poor general health (Dauria et al., 2015; Goldman, 2019; Lee et al., 2014). Beyond the individual's immediate social ties, concentrated incarceration rates at the neighborhood, zip-code, county, and state levels, have been positively associated with a range of physical and mental morbidities (Dauria et al., 2015; Escobar & Taheri, 2020; Frank et al., 2013; Hatzenbuehler et al., 2015; Holaday et al., 2021; Kajeepeta et al., 2020, 2021; Nowotny et al., 2020; Ojikutu et al., 2018; Porter, Thomas, & Emch, 2010; Topel et al., 2018), including adverse birth outcomes – the focal health outcome in this paper (Chambers et al., 2018; Conway, 2021; Dyer et al., 2019; Jahn et al., 2020; Sealy-Jefferson et al., 2020; Wallace et al., 2015, 2017; Wildeman, 2012).

Low birth weight ( ≤ 2500 g, LBW) and infant mortality occur at approximately twice the rate in Black versus White populations, and preterm birth ( ≤ 37 weeks gestational age, PTB) is nearly 35% more likely in Black compared to White women (Larrabee Sonderlund, Schoenthaler, & Thilsing, 2021). LBW and PTB are associated with increased morbidity (e.g., coronary heart disease, high blood pressure, asthma, cerebral palsy, and cognitive impairments) and mortality throughout the life course (Belbasis et al., 2016; Platt, 2014). While these trends improved throughout the 1990s and 2000s, the past decade has seen the gap continually expand with an average 2–5% change per year since 2014 (Pollock et al., 2021). Even more alarming, these disparities persist after controlling for a range of factors, including health status, education, and income. That is, Black women with the highest social status in the U.S. still fare worse than their White counterparts (Larrabee Sonderlund et al., 2021) and these disparities have widened even further during the COVID-19 pandemic (Stephenson, 2022). The literature suggests multiple structural- (e.g., health care access, poverty) and individual-level (e.g., experiences of interpersonal discrimination) factors that partly explain these disparities. Recent research, however, indicates that the noted ripple effects of increased incarceration rates in low-income Black communities represent an important, yet scarcely researched factor as well. For instance, concentrations of incarceration at the state level correlate positively with infant mortality, PTB, and LBW (Conway, 2021; Wallace et al., 2015, 2017; Wildeman, 2012). Similar associations for PTB and LBW are evident at the county (Chambers et al., 2018; Dyer et al., 2019; Jahn et al., 2020) and neighborhood levels (Sealy-Jefferson et al., 2020) as well. The evidence is mixed as to whether these relationships are different for Black versus White populations with some studies finding no between-group variation (Wallace et al., 2015, 2017) and others detecting stronger correlations in Black populations (Conway, 2021; Dyer et al., 2019; Wildeman, 2012). Regardless, given the considerable clustering of incarceration rates in low-income Black populations in underserved areas, the health consequences of incarceration will presumably be borne disproportionately by this demographic as well.

### 2.2 Spatial clustering of incarceration and community-level pathways to adverse birth outcomes

Despite the evidence of an independent correlation between spatial clusters of incarceration and community-level rates of adverse birth outcomes, few studies have examined the mechanisms that underpin this association. Fig. 2 represents theoretical pathways – derived from the literature – that may connect these two phenomena and which we have identified as requiring further empirical attention. Specifically, we pinpoint three primary pathways through which spatial clusters of incarceration lead to disparities in adverse birth outcomes (Haslam et al., 2018, p. 2018; Hux, Catov, & Roberts, 2014; Leimert & Olson, 2020; Olson et al., 2015). These relate to psychosocial stressors, community threat and victimization, and community divestment. In the following sections, we outline each of these pathways in detail before discussing implications and recommendations for future research. Given the noted overrepresentation in the prison population of Black men from low-income majority-Black neighborhoods, the health consequences of incarceration also affect these communities disproportionately. As such, we argue that racial disparities in incarceration rates represent a key driver of racial disparities in adverse birth outcomes at the population level.

#### 2.2.1 Pathway 1: social capital, community cohesion, and allostatic load

Past research has found that chronic stress and the associated allostatic load (i.e., physiological wear and tear on the body from repeated exposure to chronic stressors (Geronimus, 1992; Geronimus, 1996; Geronimus et al., 2006; McEwen & Stellar, 1993)) significantly increases the risk of adverse birth outcomes (Hux et al., 2014; Leimert & Olson, 2020; Olson et al., 2015). No studies known to the authors, have empirically examined whether spatial concentrations of incarceration contribute to community-level rates of adverse birth outcomes via stress. This mechanism nonetheless seems both plausible and likely in the context of the broader evidence base. Numerous studies indicate that the imprisonment of an individual causes debilitating and chronic stress to proliferate throughout their social network. For example, within the family unit of an incarcerated individual, financial pressure points might emerge due to legal fees, loss of a source of income, as well as the general expenses – typically borne by the family – of being incarcerated (e.g., phone calls, commissary bills, visits) (Weidner & Schultz, 2019). Financial strains often continue post release as people with a criminal record often have difficulty securing gainful employment. Many also experience post-traumatic stress or other debilitating health issues as a result of their incarceration, representing another barrier to employment and safety-net services (Alexander, 2010, p. 377). These problems may have stressful and long-term economic consequences that complicate access to basic necessities, including adequate housing, health insurance, and child care (Wildeman & Muller, 2012).

Beyond financial stressors, social networks may experience psychological burdens as well. These include not only general worry (e.g., vicarious experience of incarceration), but also the loss of any social support provided by the incarcerated individual (Weidner & Schultz, 2019). Ample research shows the crucial importance of supportive relationship ties in buffering against stress and allostatic load (Garner, 2021; Larrabee Sonderlund, Thilsing, & Sondergaard, 2019). The incarceration of someone may therefore have abrupt and severe practical and psychological repercussions for anyone (wife, parent, child, friend) relying on this individual for regular instrumental (e.g., income, childcare) and/or emotional support. Exacerbating this effect, the stigma of being connected to a person in prison might lead to internalized shame and ostracism from the broader community, further eroding existing social support systems and psychological resilience (Feingold, 2021; Tyler & Brockmann, 2017).

In addition to the loss of social support and capital, another psychological toll on family and friends may result from their regular interaction with the criminal justice system as they maintain their connection with the incarcerated. For example, visitors at prisons are typically frisked and questioned by prison guards upon arrival, and correctional officers may – and often do – pry into personal correspondence and phone calls between the incarcerated and their social connections (Comfort, 2007). Even after release, law officers are tasked with keeping tabs on parolees and in that capacity may, for instance, show up unannounced and search a paroled individual's person and residence regardless of who else might be present (parents, spouse, children, etc.). These types of situations may be experienced by friends and family of the incarcerated as denigrating and traumatizing violations of privacy and personal sovereignty (Comfort, 2007).

As such, the incarceration of an individual affects entire social networks in highly consequential ways, often causing lasting psychological trauma and stress. Importantly, the impact of these stressors is likely to be deeper and more destructive in networks that are already strained by other issues (poverty, unemployment) – as is often the case in the low-income Black communities that bear the brunt of mass incarceration (Alexander, 2010, p. 377). Integrating this with the literature on the link between stress, allostatic load, and adverse birth outcomes, it follows that the risk of birth complications increases in social networks directly affected by incarceration because of the associated stress. Given the sheer number of persons imprisoned in the U.S. (over two million on any given day, ostensibly affecting an analogous number of social networks amounting to tens of millions of people (Comfort, 2007)), it is therefore probable that incarceration contributes to population-level adverse birth outcomes via social network stress proliferation alone (Weidner & Schultz, 2019).

The evidence also suggests that the collateral effects of incarceration are far from entirely contained within the individual's immediate social network. Specifically, because incarceration rates are concentrated in certain areas, the cumulative burden of incarceration across multiple geographically proximal and potentially overlapping social networks might overflow and negatively affect people with no direct association with an incarcerated person (Comfort, 2007). This community-spillover effect probably occurs in intricate ways. Social networks that are dealing with the added stress of incarceration are likely less able to actively participate in the broader community as they preserve their time and energy to cope with their own immediate circumstances. This reduced capacity for outward solidarity might manifest as diminished instrumental support offered to others outside of the core network (e.g., helping neighbors or colleagues, contributing to neighborhood organizations). Concurrently, it probably also restricts general community participation (e.g., in faith-based and religious organizations, civic activities), limiting opportunities to provide emotional support to similar others or facilitate new social connections across networks (friend-of-a-friend connections) (Weidner & Schultz, 2019). In this way, neighborhoods affected by high-incarceration rates might become less cohesive as social networks become exceedingly disconnected from one another, eventually representing a collection of disparate individual groups rather than a unified and supportive community. This breakdown of informal social support systems and connection may lead to increased population exposure to stress as individual networks – directly affected by incarceration or not – are left to negotiate any adversity with little social capital or support from the broader community. In turn, this theoretically contributes to greater individual vulnerability to the ill effects of chronic life stressors (e.g., allostatic load) (Haslam et al., 2018, p. 2018), increasing the population risk of adverse birth outcomes along this pathway.

#### 2.2.2 Pathway 2: community crime and police victimization

Pathway 2 focuses on the extent to which concentrations of incarceration contribute to adverse birth outcomes via stress that occurs as a function of perceived risk of crime and threat of police victimization at the community-level.

While incarceration is broadly considered a deterrent to crime, the evidence on whether it actually has this effect is mixed at best. Past research has found that incarceration correlates inversely with crime, but only up to a point after which the association disappears (Clear, 2009). Further, when spatially concentrated, incarceration may even have a criminogenic effect. For example, research suggests that people who return to high-incarceration communities after serving time, saddled with a criminal record and with few legitimate prospects, may be more likely to engage in criminal activities to make ends meet (Clear, 2009). Spatial concentrations of incarceration may thus perpetuate and increase crime in a given area rather than prevent it. With studies positively correlating neighborhood crime with PTB and LBW (Mayne et al., 2018; Messer et al., 2006; Okah et al., 2014), this represents another potential mechanism by which incarceration impacts on adverse birth outcomes.

While little research has examined the specific way in which crime rates influence birth outcomes, it is probably due in part to the stress and allostatic load that results from chronic feelings of insecurity and threat of victimization throughout the community, especially among women (Clear, 2008; Weidner & Schultz, 2019). These stressors relate not only to criminal victimization, but also to the greater risk of police violence that may follow from the increased police presence in these areas. Studies on policing in low-income majority-Black neighborhoods show that community members typically feel threatened rather than reassured by police, due to frequent and historical experiences of police discrimination, harassment, and violence against Black people (Shmool et al., 2015). Notably, Krieger (Krieger et al., 2015) found that between 1960 and 2010, Black men aged 15–34 were consistently 2.5–10 times as likely as White men to die by legal intervention. Relating specifically to adverse birth outcomes, other research indicates that living in an area with a high police presence or high rates of police violence predicts PTB and particularly so among Black women (Goin et al., 2021) (Hardeman et al., 2021). Similar studies have connected police presence with stress and anxiety (Geller et al., 2014), suggesting psychophysiological mechanisms of the impact on birth outcomes.

The threat of victimization by police and/or crime may also influence birth outcomes through health-risk behaviors. For instance, research indicates that police exposure and neighborhood crime rates are positively associated with smoking (Shareck & Ellaway, 2011), substance use (Jindal et al., 2021), unprotected sex (Jindal et al., 2021), and poor sleep quality (Testa, Fahmy, & Hill, 2022), often with stronger associations for women than for men. All these behaviors are known risk factors for adverse birth outcomes (Chang et al., 2010; Fazzi et al., 2017; Hegaard et al., 2007).

In these ways, by stigmatizing neighborhoods and perpetuating rather than controlling crime, concentrated incarceration may contribute to a chronically hostile psychosocial environment that individuals navigate on a daily basis with potentially serious repercussions for maternal psychophysiological and physical health (Theall, Drury, & Shirtcliff, 2012; Topel et al., 2018).

#### 2.2.3 Pathway 3: infrastructural disinvestment

As we have argued throughout this paper, community context in terms of opportunities, resources, and constraints, may exacerbate or mitigate the effects of incarceration on community-level rates of adverse birth outcomes. As such, the third pathway centers on the stigmatization of high-incarceration areas and the community and infrastructural disinvestment that may follow. While research focused on community disinvestment and health outcomes connects less explicitly to incarceration and adverse birth outcomes, we note the well-documented unequal distribution of infrastructural and commercial resources to especially majority-Black neighborhoods that are affected by high incarceration rates and poverty (Clear, 2009; Klinenberg, 2015; Massey & Brodmann, 2014). Specifically, we argue that the social disruption, erosion of community efficacy and power, and area stigmatization caused by high incarceration rates contribute to a general pattern of government abandonment and deprivation of these areas, which may lead to a range of secondary health risks that likely impact negatively on birth outcomes (Clear, 2008; Conway, 2021; Kellogg, 2015). For example, underserved and deprived neighborhoods are often characterized by degradation of the built environment, increased air pollution and chemical exposure, few and unkempt green areas, as well as poor street lighting and walkability (Kawachi & Berkman, 2003). All these factors may increase the risk of adverse birth outcomes either directly (pollution, chemical exposure) or through psychophysiological stress reactivity (Anthopolos et al., 2014; Nwanaji-Enwerem et al., 2021). Further, commercial interests (e.g., quality grocery stores, gyms) may also be deterred from investing in these areas for fear of business failure or due to discriminatory policies such as higher mortgage and insurance costs. All of these issues affect the extent to which people can achieve optimal health (e.g., access to food, clean environment, exercise options) and thus are likely to impact on rates of adverse birth outcomes in these communities.

Transportation infrastructure and funding is less recognized as a marker of structural disadvantage but has had, and continues to have, a significant negative impact on population well-being and health, particularly in Black communities. In the case of transportation, in rural and urban areas alike, access to public transportation, remains a key indicator of health. Specifically, research has shown that lack of transportation in general can result in missed or delayed health care appointments, including for example prenatal care (Syed, Gerber, & Sharp, 2013). In contrast, adequate public transportation facilitates access to healthier foods, recreational activities, and health care (Bader et al., 2010). A significant source of funding for public transportation is linked to state and legislative actions and entwined with neighborhood and economic segregation. A case in point is the Bay Area Rapid Transit rail system and freeway systems, a critical mode of transportation that connects two major cities but has accelerated racialized residential segregation and had differential impacts on suburban vs. urban communities. For example, the rail system increased home values in the suburbs (predominantly White families), but contributed to the displacement and blight of predominantly Black families in urban areas (Golub, Marcantonio, & Sanchez, 2013).

Perhaps a more extensively studied area in community disinvestment that relates more directly to adverse birth outcomes concerns public hospital quality and closures. A recent study showed that between 2003 and 2013, 10% of obstetrical units in the U.S. closed. Closures were more likely in rural majority-Black communities and were in turn associated with increased maternal morbidity in those areas (McGregor et al., 2021). Evidence similarly suggests that the quality of health care is compromised in hospitals in low-income and high-incarceration majority-Black areas due to equipment shortages, underfunding, and high staff turnover (Janevic et al., 2020, 2021). Together, these factors limit the extent to which Black pregnant people residing in these areas can access the necessary resources to reduce the risk of birth complications.

## 3 Discussion

We have proposed a theoretical framework for how structural racism might account for racial health disparities in general, and adverse birth outcomes specifically. In line with current thinking and evidence, our framework reflects the reality that racial biases permeate society, manifesting across multiple societal systems to produce systematic and persistent population health inequalities. While this understanding of the structural nature of racism has gained traction across the health sciences in recent years, the phenomenon is seldom accounted for in scientific research. Part of the reason for this shortcoming presumably relates to the oft-cited difficulty of defining and measuring structural racism in empirical studies (Bailey et al., 2021). Our framework complements existing efforts to remedy this situation (Adkins-Jackson et al., 2021) by providing a useful roadmap for including structural racism as a key public health determinant in relevant health scientific research.

Demonstrating the utility of our framework, in the present paper we examined the association between structural racism and adverse birth outcomes as a function of incarceration. To illustrate the application of our framework, we first outlined the extent to which the systems of housing, economy, and criminal justice interact to geographically isolate, economically marginalize, and criminally target low-income Black communities. At the intersection of these three systems, we then conceptualized structural racism for the purposes of this case study as racial and economic disparities in incarceration rates. At this level of specification, and drawing on relevant research, we narrowed our focus to identify the key theoretical mechanisms that might underpin the association between spatial concentrations of incarceration (our proxy for structural racism) and adverse birth outcomes. As a result of this process, we arrived at the three pathways that we have described above, and which represent potential avenues for future research.

### 3.1 Knowledge gaps & future research

Establishing the accuracy of each of the identified mechanisms requires extensive empirical research. For example, the psychophysiological stress associated with the social breakdown described in Pathway 1, touches on multiple topics. As noted, the exchange of emotional and instrumental support within and between social networks likely represents a key buffer against stress and allostatic load and by extension adverse birth outcomes. However, ascertaining whether spatial concentrations of incarceration destabilize these systems of social support to the extent that they lose their protective resilience-building capacity requires further research. Ideally, this would include qualitative and quantitative social network analyses focusing on community disconnectedness and social isolation in high-incarceration areas, combined with longitudinal life-course studies of maternal and child psychophysiological health. Within this context, gaining knowledge about the potentially differential and/or interacting impact of distinct incarceration-related stressors that impact people in these communities (e.g., family functioning, income, housing, mental health) is also relevant – especially in terms of developing interventions or policies that target specific relief mechanisms (e.g., financial or psychological support). Finally, designing studies that tap the potential experience of stress by indirect association with an incarcerated individual – perhaps at different degrees of separation – would provide new insight into the general community impact beyond individual social networks. Insight into these mechanisms would require sociological and/or social psychological qualitative investigation.

The empirical validation of Pathway 2 and 3 similarly necessitates studies that connect the psychosocial environment in high-incarceration areas (e.g., risk of police or crime victimization) with adverse birth outcomes via health-risk behavior. This research would need to uncover how and to what extent factors in the social, commercial, and built environments of a given area facilitate or discourage healthy lifestyles, and how these influences might be shaped by local incarceration rates. Gathering this knowledge would likely require a range of different approaches. This might include cross-sectional or longitudinal mediation studies that test the relationship between resident perceptions of their neighborhood settings with specific health-risk behaviors. Ethnographic studies would also be valuable in terms of gaining qualitative insight into the lived experience in high-incarceration areas as a factor in lifestyle and health behavioral choices and options. Examining public and commercial investment motivation, behavior, and (dis)incentives in high-incarceration areas would likewise be pertinent information in terms of connecting incarceration rates with the lack or presence of healthy or unhealthy features in the built environment. These may include greenery, exercise facilities, food deserts, and health care accessibility.

While elucidating these pathways would represent valuable knowledge, we highlight the general scarcity of detailed datasets on incarceration and population health as a key barrier to accomplish this end. To our knowledge, there are only few large-scale longitudinal datasets that contain information on incarceration and health outcomes, including the Longitudinal Study of Adolescent Health, the Fragile Families & Child Wellbeing study, and the National Longitudinal Survey of Youth. Studies in this area, however, have also pulled data from multiple other sources to investigate associations between spatial concentrations of incarceration and community-level health, including adverse birth outcomes. Conway (Conway, 2021), for instance, linked incarceration data from US Bureau of Justice Statistics reports with data from KIDS COUNT data center and the CDC's WONDER database to analyze the impact of state-level incarceration rates on adverse birth outcomes. Other sources of publicly available and detailed spatial incarceration data are maintained by non-profit organizations, including the Vera Institute and the Prison Policy Initiative. Not only could accessing these data shed light on long-standing concerns about mass incarceration and health outcomes, but it also provides an opportunity to build partnerships and alliances to identify and co-develop community-driven solutions to promote health equity and justice.

### 3.2 Policy and community-level interventions

Disrupting the identified pathways that lead from spatial clustering of incarceration to adverse birth outcomes may be achieved at the social network as well as the community levels. However, we would be remiss if we did not first note the policy-level changes that might address the root of the problem by focusing on curtailing the excessive rate of incarceration and eliminating its racialized elements. Two factors wholly determine the size of the prison population at any given time: The number of people who are sent to prison and the length of time they are sentenced to stay there (Frost, 2006). Decreasing either or both of these numbers, and thus reducing the prison population, would presumably also lessen the community health repercussions of incarceration described above (Wildeman & Wang, 2017). This may be realized by reforming key criminal justice policies that drive mass incarceration and impact minority populations disproportionately. These include mainly tough-on-crime policing strategies (e.g., stop-and-frisk, reasonable suspicion) and harsh sentencing guidelines for non-violent crimes (e.g., three strikes laws, mandatory minimum sentencing) (Clear, 2009). Some of these changes have already been attempted with promising results, including legalization of particular drugs and expansion of probationary sentencing in place of incarceration (Grucza et al., 2018). Other trials have included restructuring and refocusing police strategies away from aggressive and punitive tactics and towards collaborative and preventive approaches. For example, evaluations of community policing practices – where police work alongside community members to prevent and deal with crime constructively – have generated encouraging results in cities and urban areas. In Chicago, community policing was associated with improvements in informal social control outcomes as well as community attitudes towards police (Lombardo & Donner, 2018). Similar research found positive effects on community trust and perceptions of police legitimacy (Peyton, Sierra-Arévalo, & Rand, 2019).

Outside of implementing policy changes in the criminal justice system, other approaches might involve equipping those areas most affected by incarceration with the resources needed to mitigate the associated community health consequences. For example, in terms of adverse birth outcomes, past research has found that the quality of hospital of delivery accounts for a considerable portion of racial disparities in this outcome. Specifically, hospitals closest to low-income majority-Black neighborhoods (which are often most at risk of incarceration) are often underfunded and understaffed and therefore limited in their capacity to provide adequate prenatal and obstetric care, increasing the risk of pregnancy and birth complications (Howell & Zeitlin, 2017; Janevic et al., 2020, 2021). Providing better funding for local health care facilities in high-incarceration areas might therefore contribute considerably to narrowing the racial gap in adverse birth outcomes and bringing down population-level incidence rates.

Another strategy that focuses more on supporting individuals and their families could include the Earned Income Tax Credit (EITC)—a refundable tax credit for low-income workers. The policy aims to both reduce poverty and encourage work by providing a refundable credit at tax time to eligible low-income families (who are also most affected by incarceration). Though the program has been in existence since 1975, only few studies (e.g., the CDC's HI-5 initiative (CDC, 2022)) have examined the associated health impacts. The results have been positive including improving infant birth weight and reducing health-risk behaviors such as smoking.

In addition to policy and resource-based solutions, there are also promising community-level interventions and initiatives. Given the central role of stress in the identified pathways that link spatial concentrations of incarceration with adverse birth outcomes, efforts to reduce environmental stressors and boost resilience in communities affected by high incarceration rates are likely to have a positive impact in this context. Supporting this idea, research indicates that community-led initiatives that focus on providing pre- and postnatal emotional, psychological, and practical support to Black women and their families reduce the risk of adverse birth outcomes in this population. Examples of such efforts include the Black Infant Health Program and the JJ Way. Both of these initiatives, and others like them, have been shown to improve racial disparities in adverse birth outcomes by supporting Black women through group trainings on stress management, building social support, and pre- and postnatal health and well-being (Josephs and. E, 2017; Nichols & Cohen, 2021).

Finally, reintegration interventions for justice-involved individuals including job training, work programs, and free formal education, could strengthen not only individuals, but also communities. Reinstating voting rights and allowing for the expungement of individual's criminal history (e.g., for misdemeanor crimes) also represent key issues in successful reintegration (Clear, 2009; Morenoff & Harding, 2014). Similarly, support and trainings that focus on helping individuals repair relationships with their social network could have an indirect impact on communities and support community resilience (Andersen et al., 2020). Reintegration is not an easy process, but community-led approaches that focus on civic responsibility (i.e. voting, volunteerism (Morenoff & Harding, 2014)) and are tailored to specific populations (e.g. older offenders, women) are necessary components that could have lasting impacts on communities.

### 3.3 Limitations

There are a few limitations to the present paper. While we focus on the implications of structural racism for minority health outcomes in the U.S., we note that the phenomenon of structural racism by no means is unique to this country and is evident in many White-dominant societies around the world, including Canada (Tuyisenge & Goldenberg, 2021), Australia (Plater et al., 2020), New Zealand (Came, Baker, & McCreanor, 2021), and numerous European countries (Miller, 2021; Razai, Majeed, & Esmail, 2021). This may come as no surprise as these nations and regions were built on much of the same European colonial history as the U.S. (Painter, 2010). Expanding on this point, we also note that structural racism and discrimination does not only impact on Black populations, but also affects other minorities defined by race, ethnicity, nationality, gender, sexual orientation, and more. For other populations, however, the health impact of structural racism may originate in societal domains other than those listed here and operate along different pathways as well. For example, given the history of Black-White segregation in the U.S., redlining might be a particularly relevant manifestation of structural racism as it relates to Black populations in this country. Shifting the focus to, say, immigrants in the U.S., however, may instead require a detailed analysis of U.S. immigration policy, border patrol, and immigrant rights. Thus, in structural racism research, acknowledging the unique history of the particular group(s) in focus is imperative to understand the various ways in which discrimination against the given demographic is enacted systematically within and across societal institutions and systems.

## 4 Conclusion

In the present paper, we propose a comprehensive framework for the association between structural racism and health outcomes. The framework is intended as a guide for future investigation in this area, providing a starting point for generating research questions and hypotheses. Demonstrating the utility of our framework to this end, we applied it to the link between spatial racialized clustering of incarceration and community-level racial disparities in adverse birth outcomes. Within this context we identified and described three key pathways that might explain the association between our variables of interest. Ultimately, these pathways highlight the fact that while imprisonment may be intended as individual punishment, it often has dire social, psychological, and health consequences for the social network and community in which the individual exists. Further, these spillover effects appear to intensify exponentially as incarceration rates increase and cluster geographically. In light of this, we argue that spatial and racialized concentrations of incarceration should be considered a potential community-wide risk factor for a range of negative health outcomes, including birth and pregnancy complications. Efforts to lower the population-level risk of adverse birth outcomes should therefore include a special focus on mass incarceration and high-incarceration areas. To verify this contention, we encourage any research efforts to test the identified pathways to ascertain whether incarceration, as a function of structural racism, may provide an explanation for some of the observed population-level differences in adverse maternal health outcomes. Finally, while uncovering each of these pathways will likely provide added insight into the mechanics of the relationship between spatial concentrations of incarceration and birth complications, we note that this outcome is likely impacted by structural racism through multiple other pathways unrelated to incarceration. Some of these likely originate in other systems (e.g., education, environmental pollution) than the ones we identified here and thus require separate attention.

## Ethical statement

Mia Charifson is supported by the 10.13039/100000001National Science Foundation Graduate Research Fellowship Program (RN Grant ID: 20-A0-00-1005789). This sponsor had no active role or influence in this research, including in terms of study design; in the collection, analysis and interpretation of data; in the writing of the report; and in the decision to submit the article for publication Any opinions, findings, and conclusions or recommendations expressed in this material are those of the authors and do not necessarily reflect the views of the National Science Foundation. None of the other authors received any funding towards this particular paper.

## Funding sources

MC is supported by the 10.13039/100000001National Science Foundation Graduate Research Fellowship Program (RN Grant ID: 20-A0-00-1005789). Any opinions, findings, and conclusions or recommendations expressed in this material are those of the authors and do not necessarily reflect the views of the National Science Foundation.

## Declarations of interest

None.

## Data Availability

No data was used for the research described in the article.
